# Clustering of behavioral and chronic health risk factors and their association with self-reported health and cardiovascular disease outcome among adults in North Carolina

**DOI:** 10.3934/publichealth.2025035

**Published:** 2025-07-02

**Authors:** Chukwuemeka E Ogbu, Stella C Ogbu, Maureen Ezechukwu, Sushma Lamsal, Ifeanyi I Momodu

**Affiliations:** Department of Internal Medicine, Cape Fear Valley Health, Fayetteville, NC, United States

**Keywords:** cardiovascular disease, health behaviors, latent class analysis, chronic disease, mental health, public health disparities

## Abstract

**Background:**

In 2024, North Carolina (NC) had a smoking rate of 17.2% and a higher-than-average rate of binge and heavy drinking. These behaviors often cluster with other health risks such as hypertension, hypercholesterolemia, and diabetes, thus leading to significant disparities in cardiovascular, physical, and mental health outcomes across the state. However, limited research has examined these clustering patterns within North Carolina.

**Objective:**

This study seeks to investigate the associations between latent class membership, defined by clustering of behavioral and chronic health risk factors, and cardiovascular disease, self-reported health status, physical health status, and mental health status.

**Methods:**

We conducted a cross-sectional analysis using the 2017, 2019, and 2021 North Carolina Behavioral Risk Factor Surveillance System (BRFSS) data. A latent class analysis (LCA) was used to identify distinct health risk profiles among adults based on smoking, alcohol use, physical activity, fruit and vegetable intake, hypertension, elevated cholesterol, and diabetes status. Multivariable logistic regression models were used to examine associations between latent class membership and four outcomes: cardiovascular disease (CVD), self-reported general health, physical health status, and mental health status. Analyses were adjusted for sociodemographic variables, and age-stratified analyses were conducted.

**Results:**

The LCA identified two distinct classes: “Moderate drinking overweight non-smokers” (Class 1) and “High behavioral and chronic risk profile” (Class 2). Class 1 was characterized by moderate alcohol consumption, overweight status, and low smoking prevalence, while Class 2 reflected a higher prevalence of smoking, binge drinking, hypertension, diabetes, and elevated cholesterol. Membership in Class 2 was significantly associated with increased odds of CVD (OR = 1.93; 95% CI: 1.60–2.34), poor self-reported health (OR = 1.69; 95% CI: 1.46–1.96), ≥14 days of poor physical health (OR = 1.82; 95% CI: 1.55–2.15), and ≥14 days of poor mental health (OR = 1.68; 95% CI: 1.43–1.97). In age-stratified analyses, the strongest associations were observed among young adults (18–39 years), with significantly higher odds of CVD (OR = 6.84; 95% CI: 2.79–16.72), poor physical health (OR = 2.32; 95% CI: 1.58–3.40), and poor mental health (OR = 2.12; 95% CI: 1.60–2.81). Similar but attenuated associations were observed among adults aged 40–59 and ≥60 years.

**Conclusion:**

These findings support the importance of targeted public health efforts in North Carolina that address the co-occurrence of behavioral and chronic health risk factors, especially among younger populations. Syndemic-informed interventions which focus on behavioral and proximal chronic disease risk factors may help reduce CVD burden and improve the population health.

## 1. Introduction

Health risk behaviors such as tobacco use, excessive alcohol consumption, poor dietary intake, and physical inactivity have long been recognized as significant contributors to the growing burden of chronic diseases in the United States [1],[2]. These behaviors increase the risk of developing cardiovascular disease (CVD) and have been associated with poorer physical and mental health outcomes [3]–[5]. Cardiovascular disease remains the leading cause of death in the U.S., accounting for nearly 702,880 deaths in 2022, which is equivalent to one in 5 deaths [6]. The cost of healthcare services, medicines, and loss of productivity due to death from CVD was estimated to be above $250 billion from 2019 to 2020 [7]. In North Carolina, more than 800,000 adults, representing 10.3% of the adult population, have reported a history of heart attack, angina, or stroke. CVD remains the leading cause of hospitalization in the state, accounting for over 155,000 hospital discharges and 20% of all deaths in 2018 [8],[9].

While individual health behaviors have often been independently studied, evidence increasingly suggests that these behaviors tend to co-occur and cluster within populations [10]–[12]. Individuals who smoke may also be more likely to excessively consume alcohol or engage in poor dietary practices, while those who are physically inactive may have an elevated risk for multiple chronic conditions [2],[12]. Moreover, these clusters of risk behaviors can have a cumulative effect and increase susceptibility to poor self-reported health and physical and mental health outcomes [13],[14]. In North Carolina, 23% of adults report no engagement in leisure-time physical activity, 17% report current cigarette smoking, 32% are obese, and 18% report binge or heavy drinking [15]. In addition, dietary behaviors remain suboptimal, with only 10.4% of adults consuming fruit and 8.1% consuming vegetables one or more times per day [16].

Beyond behavioral risk factors, the burden of chronic health conditions such as hypertension, diabetes, and high cholesterol remains high [17]–[19]. These conditions often develop as downstream consequences of persistent unhealthy behaviors, but may also shape future health risks and behaviors, thus creating a cycle of poor health over time [20]–[22]. However, despite their clear interrelationship, health behaviors and chronic conditions have traditionally been studied as separate domains. This approach may overlook important interactions between these factors and how they combine to influence population health [23],[24].

Recent scholarship has adopted a syndemic framework to better understand these complex interrelationships. The syndemic theory posits that multiple health conditions and risk behaviors co-occur, interact, and exacerbate one another within specific populations, which are often shaped by broader social, economic, and structural factors [25]–[28]. From this perspective, health behaviors and chronic conditions are not isolated phenomena but components of intertwined health risks that collectively worsen the health outcomes, particularly among socioeconomically disadvantaged groups.

Identifying how behavioral risk and chronic disease risk factors cluster within populations is important to inform effective public health interventions. A latent class analysis (LCA) is a statistical method that allows one to identify distinct subgroups based on patterns of health-related characteristics [29]. Previous studies have used an LCA to examine the clustering of health behaviors [13],[30]–[34], while others have used chronic disease diagnoses to capture chronic health risk profiles [35]–[37]. However, few studies have applied this approach to examine how both behavioral and chronic health risk factors are simultaneously clustered within a U.S. state population.

The health status of adults in North Carolina further illustrates the relevance of this approach. Nearly 18% of adults in the state report fair or poor general health, with an average of 3.6 physically unhealthy days and 4.1 mentally unhealthy days each month [15]. These indicators, which are drawn from the Behavioral Risk Factor Surveillance System (BRFSS), reflect population-level perceptions of functional health and psychological well-being. “Physically unhealthy days” refer to days in the past month when physical health was “not good” due to illness or injury, while “mentally unhealthy days” reflect periods affected by stress, depression, or emotional difficulties [38].

Therefore, the aim of our study is to identify latent classes of North Carolina adults based on the clustering of behavioral and chronic health risk factors and to examine the associations of these latent classes with four key health outcomes: self-reported general health, physical health status, mental health status, and cardiovascular disease. We hypothesize that distinct latent classes will emerge, and these risk profiles will be associated with poorer outcomes across all four domains.

## 2. Materials and methods

We used the 2017, 2019, and 2021 North Carolina Behavioral Risk Factor Surveillance System (NCBRFSS) for this analysis [39]. Our dataset consisted of 14,139 participants who were 18 years or older. The NCBRFSS is a state-based surveillance system that collects information on preventive health practices, health risk behaviors, healthcare access, and utilization related to chronic disease and injuries and preventable infectious diseases. Trained survey administrators identified respondents using random-digit-dialing methods, and conducted surveys via landline and cell phone interviews [39]. The BRFSS employs a complex sampling design that adjusts the respondent data to known proportions of age, race, ethnicity, and sex within the state [39]. In 2017, North Carolina's combined landline and cell phone response rate was 35.9 percent [40]. The combined response rates were 40.8 in 2019 and 43.4 in 2021 [41],[42]. The response rates were calculated using standards set by the American Association of Public Opinion Research Response Rate formula [43]. The NCBRFSS received approval from the States' Institutional Review Boards (IRB) and the North Carolina Division of Public Health Ethics Review Board, thus instilling confidence in the study's ethical standards. The NCBRFSS adheres to a stringent ethical process to protect the participants and ensure data integrity. The participants provided informed consent, were assured of confidentiality with personal identifiers removed, and voluntarily participated. The survey's methodology and procedures underwent review by an IRB to meet ethical standards and federal regulations. Data security measures were implemented to secure the collected data and restrict access [39]. This study is exempt from the full IRB process because the BRFSS is a public-use dataset. A detailed description of the data, sampling method, and other analytical guidelines is available elsewhere [40].

### 2.1. Assessments

#### 2.1.1. Demographics variables

The participants provided demographic information, including age, sex, race/ethnicity, educational level, and income. Age was categorized into 18–39 years, 40–59 years, and 60+ years. Sex was recorded as male or female, while race/ethnicity was classified into White non-Hispanic, Black non-Hispanic, Hispanic, or multiracial/other non-Hispanic categories. Educational attainment was classified into four categories: did not graduate high school, graduated high school, attended college or technical school, and graduated from college or technical school. Income was categorized as less than $15,000, $15,000 to <$25,000, $25,000 to <$35,000, $35,000 to <$50,000, $50,000 to <$100,000, $100,000 to <$200,000, and $200,000 or more.

#### 2.1.2. Assessment of health-related risk factors

Health-related factors were assessed using the NCBRFSS self-administered questionnaire as follows:

• **Hypertension:** Hypertension was defined as a self-reported diagnosis of high blood pressure or having been told by a healthcare professional that they have hypertension. The responses were categorized as “yes” or “no”.

• **Diabetic status:** Diabetic status was based on the participants' reports of being told by a healthcare professional that they had diabetes. The responses were categorized as “yes” or “no”. Those who reported gestational diabetes or prediabetes/borderline diabetes were coded as missing. Gestational diabetes and prediabetes were excluded to maintain a clear distinction between confirmed diabetes and non-disease states to minimize misclassification [44].

• **Overweight:** Overweight was defined as having a body mass index (BMI) greater than 25, which was calculated from self-reported height and weight.

• **Physical activity status:** The physical activity status was assessed using the following questionnaire: “During the past month, other than your regular job, did you participate in any physical activities or exercises such as running, calisthenics, golf, gardening, or walking for exercise?” The responses were categorized as a binary “yes” or “no” response.

• **Drinking status:** Alcohol use was based on whether the participants reported consuming at least one drink in the past 30 days. The responses were categorized as “yes” or “no”.

• **Binge drinking:** Binge drinking was defined as consuming five or more drinks on one occasion for men and four or more drinks for women. The responses were categorized as “yes” or “no”.

• **Lifetime smoking:** Lifetime smoking was determined by asking the participants whether they had smoked at least 100 cigarettes in their lifetime. The responses were categorized as “yes” or “no”.

• **Current smoking:** Current smoking status was classified into three categories based on the BRFSS data: participants who had smoked at least 100 cigarettes and currently smoked every day or some days were classified as current smokers; those who smoked 100 cigarettes but no longer smoked were classified as former smokers; and those who had never smoked 100 cigarettes were classified as never smokers.

• **Fruit consumption:** Fruit consumption was determined based on whether the participants reported consuming fruit one or more times per day. The responses were categorized as “one or more times per day” or “less than once per day”.

• **Vegetable consumption:** Vegetable consumption was assessed by asking the participants if they consumed vegetables one or more times per day. The responses were categorized as “one or more times per day” or “less than once per day”.

#### 2.1.3. Health outcomes

• **Self-reported general health:** The participants were asked, “Would you say that, in general, your health is...”. The Likert responses were excellent, very good, good, fair, and poor. The participants were categorized as “excellent/very good/good health” or “fair/poor health”.

• **Physical health status:** Physical health was assessed using the BRFSS question, “In the past 30 days, for how many days was your physical health not good?” The responses were categorized as 0 days, 1–13 days, or 14 or more days.

• **Mental health status:** Mental health status was assessed using the BRFSS question, “In the past 30 days, for how many days was your mental health not good?” The responses were categorized as 0 days, 1–13 days, or 14 or more days. The CDC Health Days questionnaire is a validated tool that is consistently used to assess the physical and mental health status and the Health-Related Quality of Life in adult populations [38].

• **Cardiovascular disease status:** Cardiovascular disease status was determined by the question, “Has a doctor or health professional ever told you that you had coronary heart disease, angina, or myocardial infarction?” The responses were categorized as “yes” or “no”.

### 2.2. Statistical analyses

The NCBRFSS uses a stratified, multistage, complex survey design to improve the representativeness of North Carolina's adult population. We followed the BRFSS analytical guidelines and applied the recommended sampling weights to account for stratification, clustering, and primary sampling units (PSUs).

First, we conducted descriptive analyses to summarize the sample characteristics and risk factor prevalence. Rao-Scott chi-square tests were used to examine the bivariate associations between the sociodemographic variables, risk factors and health outcomes. The prevalence of each risk factor (hypertension, diabetic status, overweight or obese, physical activity status, drinking, smoking, and fruit and vegetable consumption) was reported.

An LCA was conducted to identify subgroups of individuals with similar patterns of behavioral and chronic health risk factors. Models with one to six classes were estimated. This method produces two key parameters: (1) posterior class probabilities, which indicate the likelihood of each participant belonging to a specific class; and (2) item-class probabilities, which reflect the likelihood of a particular behavior being endorsed within each latent class [29]. A null model was first fitted without grouping the variables and covariates to understand the overall subclass structure. Then, these models were checked for model fitness, parsimony, and interpretability of the LCA solutions [29]. The final model was assessed by the number of optimal parameters, the Bayesian Information Criterion (BIC), the sample size Adjusted Bayesian Information Criteria (ABIC), the Akaike Information Criterion (AIC), the adjusted Lo-Mendell-Rubin adjusted likelihood ratio test (LMR-LRT), entropy, and the interpretability of the latent class solutions [29],[45]. Lower AIC/BIC and higher entropy values indicated a better model fit. A significant LMR-LRT (p < 0.05) was interpreted as evidence that the model with n classes fit better than the model with n-1 classes. Posterior class probabilities and item-response probabilities were examined for interpretability. Risk factors with response probabilities >0.50 were considered “endorsed”, and latent classes were descriptively named based on patterns of high or low endorsement [29].

An exploratory analysis was done using age, sex, and race as a grouping variable to test for measurement invariance [46]. Then, we estimated parameters when measurement invariance by age, sex, or race was imposed and evaluated their statistical significance. We found that measurement invariance holds across sex and race/ethnicity but does not hold across age groups, which suggests that the way the latent classes are interpreted varies across age groups. Consequently, all outcome models were stratified by age. Finally, we used multivariable regression models to examine associations between latent class membership and health outcomes, thereby adjusting for confounders. A binary multilogistic regression was used for CVD and self-reported general health. A multinomial logistic regression was used for the physical and mental health status. Age was not included as a covariate in the age-stratified models. The odds ratios (ORs) and 95% confidence intervals (CIs) were reported for all models. Analyses were conducted using R (poLCA package) and Mplus version 7.

## 3. Results

### 3.1. Characteristics of NC BRFSS participants by sociodemographic, health outcome, and health-related risk factors

A total of 14,137 participants were included in the analysis, with a nearly even distribution of males (48%) and females (52%) (Table 1). The participants were categorized into the following age groups: 18–39, 40–59, and 60 years and older. The largest proportion of participants (37.6%) were in the youngest age category, while 31.9% were aged 40–59, and 30.5% were 60 years or older. Most participants (65.3%) identified as White non-Hispanic, followed by Black non-Hispanic (20.8%), Hispanic (8.3%), and smaller proportions identified as either other or multiracial. The income levels considerably varied, with 41.2% of participants reporting annual earnings between $50,000 and $100,000, approximately 8.5% of participants earned less than $15,000, and fewer than 2% reported $200,000 or more earnings. 32.6% of participants attended some college or technical school, while 27.5% had completed a college degree or higher. In contrast, 13.1% had not graduated from high school. Most participants (82.1%) rated their general health as good or better, while 17.9% reported their health as fair or poor. About 67.8% of NC adults reported zero days of poor physical health in the past 30 days, while 19.7% experienced 1–13 days, and 12.5% experienced 14 or more days. Similarly, 64.9% of participants reported zero days of poor mental health, with 21.7% reported 1–13 days and 13.4% experienced 14 or more days of poor mental health. A small proportion (6.8%) reported a history of myocardial infarction or coronary heart disease. Hypertension was prevalent in the sample, with 34.9% of participants reporting the condition. About 68% of NC adults were categorized as overweight or obese based on their BMI. 16.7% of participants were current smokers, 25.1% were former smokers, and 58.2% had never smoked. Binge drinking was reported by 14.7% of participants, while 85.3% did not engage in binge drinking. Dietary and lifestyle behaviors were generally positive, with 61.4% of participants consuming fruit at least once daily and 83.1% consuming vegetables at least once daily. Three-quarters of participants (75.3%) reported engaging in physical activity or exercise during the past 30 days, while 24.7% reported no physical activity. Alcohol consumption was reported by 49.4% of participants, and 41.8% of participants endorsed smoking at least 100 cigarettes in their lifetime.

**Table 1.** Weighted sample characteristics of participants in NCBRFSS.

**Variable**	**Frequency (weighted percentage)**	**P Value**
**Sociodemographic variables**		
**Sex**		0.0002
Male	6507 (48.0)	
Female	7630 (51.9)	
**Age categories**		<0.0001
18–39 years	3969 (37.6)	
40–59 years	4510 (31.9)	
60 years and above	5414 (30.5)	
**Race/ethnicity**		<0.0001
White only, non-Hispanic	9002 (65.3)	
Black only, non-Hispanic	2719 (20.8)	
Other race only, Non-Hispanic	724 (3.8)	
Multiracial, non-Hispanic	216 (1.8)	
Hispanic	1239 (8.3)	
**Income**		<0.0001
Less than $15,000	1014 (8.5)	
$15,000 to <$25,000	1735 (15.7)	
$25,000 to <$35,000	1342 (12.2)	
$35,000 to <$50,000	1561 (14.0)	
$50,000 to <$100,000	4519 (41.2)	
$100,000 to <$200,000	745 (6.5)	
$200,000 or more	217 (1.9)	
**Educational status**		<0.0001
Did not graduate high school	1373 (13.1)	
Graduated high school	3420 (26.8)	
Attended college or technical school	3939 (32.6)	
Graduated from college or technical school	5355 (27.5)	
**Health outcome variables**		
**Self-reported general health**		<0.0001
Good or better health	11,418 (82.1)	
Fair or poor health	2671 (17.9)	
**Physically unhealthy days**		<0.0001
Zero days	9237 (67.8)	
1–13 days	2781 (19.7)	
14+ days	1840 (12.5)	
**Mentally unhealthy days**		<0.0001
Zero days	9302 (64.9)	
1–13 days	2875 (21.7)	
14+ days	1742 (13.4)	
**Cardiovascular disease status (MI/CHD)**		<0.0001
Yes	1149 (6.8)	
No	12,861 (93.2)	
**Health-related risk factors**		
**Hypertension**		<0.0001
No	8497 (65.1)	
Yes	5599 (34.9)	
**High cholesterol**		<0.0001
No	7551 (66.5)	
Yes	4722 (33.5)	
**Diabetes**		<0.0001
No	11,756 (87.7)	
Yes	1947 (12.3)	
**Overweight or Obese**		<0.0001
No	3936 (31.6)	
Yes	8896 (68.4)	
**Smoking status**		<0.0001
Current smokers	2098 (16.7)	
Former smokers	3648 (25.1)	
Never smokers	7950 (58.2)	
**Binge drinker**		<0.0001
No	11,330 (85.3)	
Yes	1767 (14.7)	
**Fruit consumption ≥1 time/day**		<0.0001
Yes	8136 (61.4)	
No	4849 (38.6)	
**Vegetable consumption ≥1 time/day**		<0.0001
Yes	10,599 (83.1)	
No	2095 (16.9)	
**Any physical activity in past month**		<0.0001
Yes	10,202 (75.3)	
No	3454 (24.7)	
**At least one drink of alcohol in the past 30 days**		<0.0001
Yes	6463 (49.4)	
No	6764 (50.6)	
**Smoked at least 100 cigarettes in entire life**		<0.0001
Yes	5755 (41.8)	
No	7950 (58.2)	

### 3.2. Sample characteristics stratified by age categories

The younger participants (18–39 years) had the highest proportion of binge drinkers (58.6%) and reported good or better general health (41.1%) compared to other age groups (Table 2). Additionally, they reported the highest levels of physical activity, with 40.1% engaging in exercise. The younger adults had the highest proportion of individuals who consumed alcohol in the past 30 days (43.4%) and were current smokers (40.2%).

The participants aged 40–59 had the largest proportion of individuals who had completed college or technical school (36.4%). 33.4% of those aged 40–59 years reported consuming alcohol in the past 30 days, and 35.1% were overweight or obese. 38.6% of adults in this group reported experiencing 1–13 days of poor physical health in the past month.

The older adults (60 years and above) had the highest prevalence of hypertension (53.9%), high cholesterol (50.2%), and diabetes (34.5%). The prevalence of MI/CHD was highest in this age group (65.9%). About 37% of the older participants were former smokers, and 36.1% engaged in regular physical activity. Dietary habits remained relatively consistent across age groups, with the older participants reporting similar fruit (31.3%) and vegetable (29.8%) consumption compared to the younger age groups.

### 3.3. Model selection

Table 3 presents the results of the fitting latent class models, which identified two distinct classes to best explain the patterns of health risk behaviors among the participants. Several key diagnostics support our conclusion: it has a significantly lower AIC and BIC than the 1-latent cluster model, thus indicating better model fit. The LMR-LRT for the 2-cluster model shows a highly significant improvement over the 1-cluster model, and the entropy value of 0.971 suggests a high level of classification accuracy. Additionally, the two-class model was more interpretable regarding the patterns of risk. While models with more clusters (3 to 6) continued to improve in terms of the AIC, BIC, and SAIC values, the improvements were marginal and were not interpretable in the observed patterns. Furthermore, the increase in model complexity is notable in these models, thus making the 2-cluster model the most parsimonious.

**Table 2.** Sociodemographic, health outcomes and health-related risks stratified by age categories.

**Variables**	**Total**	**18–39 years**	**40–59 years**	**≥60 years**	**P value**
**Sociodemographic variables**					
**Sex**					0.0001
Male	6403	1964 (38.7)	2127 (32.9)	2312 (28.4)	
Female	7488	2005 (36.6)	2381 (31.0)	3102 (32.5)	
**Race/ethnicity**					<0.0001
White only, non-Hispanic	8888	2185 (32.4)	2724 (31.4)	3979 (36.2)	
Black only, non-Hispanic	2683	721 (37.2)	976 (35.8)	966 (27.0)	
Other race only, Non-Hispanic	709	250 (54.4)	229 (29.4)	230 (16.2)	
Multiracial, non-Hispanic	214	87 (55.9)	86 (32.5)	39 (11.6)	
Hispanic	1223	669 (6.9)	437 (7.6)	117 (5.5)	
**Income**					<0.0001
Less than $15,000	1008	246 (34.2)	351 (33.6)	409 (32.3)	
$15,000 to <$25,000	1726	494 (39.3)	508 (27.3)	724 (33.4)	
$25,000 to <$35,000	1332	442 (42.2)	356 (25.3)	534 (32.5)	
$35,000 to <$50,000	1552	500 (40.9)	436 (27.4)	616 (31.7)	
$50,000 to <$100,000	4487	1267 (35.9)	1721 (39.1)	1499 (24.9)	
$100,000 to <$200,000	734	196 (32.0)	329 (44.8)	209 (23.1)	
$200,000 or more	215	63 (36.2)	105 (46.8)	47 (17.0)	
**Educational status**					<0.0001
Did not graduate high school	1358	333 (32.2)	464 (31.2)	561 (36.1)	
Graduated high school	3381	989 (39.0)	1041 (30.2)	1351 (30.8)	
Attended college or technical school	3881	1135 (39.1)	1181 (29.7)	1565 (31.2)	
Graduated from college or technical school	5245	1503 (36.8)	1815 (36.4)	1927 (26.8)	
**Health outcome variables**					
**Self-reported general health**					<0.0001
Good or better health	11,204	3569 (41.1)	3562 (30.9)	4073 (27.9)	
Fair or poor health	2642	393 (21.9)	934 (36.2)	1315 (41.8)	
**Physically unhealthy days**					<0.0001
Zero days	9054	2784 (39.9)	2916 (31.4)	3354 (28.6)	
1–13 days	2747	902 (42.2)	873 (30.7)	972 (27.1)	
14+ days	1825	239 (18.9)	665 (38.6)	921 (42.6)	
**Mentally unhealthy days**					<0.0001
Zero days	9121	2119 (31.2)	2882 (32.2)	4120 (36.5))	
1–13 days	2834	1223 (52.7)	928 (29.9)	683 (17.3)	
14+ days	1725	565 (43.0)	643 (34.5)	517 (22.5)	
**Cardiovascular disease status (MI/CHD)**					<0.0001
Yes	1137	43 (5.9)	261 (28.2)	833 (65.9)	
No	12,634	3913 (40.1)	4218 (32.2)	4503 (27.7)	
**Health-related risk factors**					
**Hypertension**					<0.0001
No	8339	3464 (50.8)	2816 (31.2)	2059 (18.0)	
Yes	5514	493 (12.8)	1681 (33.2)	3340 (53.9)	
**High cholesterol**					<0.0001
No	7392	2403 (40.7)	2638 (34.9)	4135 (24.3)	
Yes	4663	425 (13.4)	1497 (34.3)	2351 (52.3)	
**Diabetes**					<0.0001
No	11,541	3788 (42.2)	3764 (31.5)	3989 (26.3)	
Yes	1925	88 (6.9)	599 (4.5)	1238 (58.6)	
**Overweight or Obese**					<0.0001
No	3888	1364 (45.8)	1009 (25.1)	1515 (29.2)	
Yes	8816	2185 (32.8)	3094 (35.1)	3537 (32.1)	
**Smoking status**					<0.0001
Current smokers	2084	640 (40.2)	859 (38.3)	585 (21.6)	
Former smokers	3605	569 (21.5)	1037 (31.9)	1999 (46.6)	
Never smokers	7796	2635 (43.7)	2467 (30.0)	2694 (26.3)	
**Binge drinker**					<0.0001
No	11,143	2738 (33.4)	3585 (32.1)	4820 (34.5)	
Yes	1758	911 (58.6)	571 (30.7)	276 (10.8)	
**Fruit consumption ≥1 time/day**					0.0695
Yes	8009	2214 (37.1)	2544 (31.6)	3251 (31.3)	
No	4780	1409 (37.4)	1638 (33.5)	1733 (29.0)	
**Vegetable consumption ≥1 time/day**					0.0953
Yes	10,442	2964 (37.0)	3463 (33.2)	4015 (29.8)	
No	2067	593 (38.4)	676 (30.1)	798 (31.5)	
**Any physical activity in past month**					<0.0001
Yes	10,017	3091 (40.1)	3197 (30.9)	3729 (28.9)	
No	3415	715 (28.8)	1158 (35.0)	1542 (36.1)	
**At least one drink of alcohol in the past 30 days**					<0.0001
Yes	6393	2223 (43.4)	2190 (33.4)	1980 (23.2)	
No	6634	1476 (31.4)	2009 (30.4)	3149 (38.3)	
**Smoked at least 100 cigarettes in entire life**					<0.0001
Yes	5698	1210 (28.9)	1900 (34.5)	2588 (36.6)	
No	7796	2635 (43.7368)	2467 (30.0)	2694 (26.2)	

**Table 3.** Table of model selection and diagnostics.

**# of Latent clusters**	**Loglikelihood**	**Best H0 Replicated**	**# Parameters**	**AIC**	**BIC**	**SAIC**	**LMR-LRT**	**Entropy**
1	−89636.412	Yes	12	179296.823	179387.504	179349.369	N/A	N/A
2	−80214.515	Yes	25	160479.030	160667.947	160588.499	19229.454 (p ≤ 0.00001)	0.971
3	−78763.773	Yes	38	157603.546	157890.700	157769.939	2878.316 (p ≤ 0.0001)	0.840
4	−77636.757	Yes	51	155375.515	155760.906	155598.833	2236.033 (p ≤ 0.0001)	0.809
5	−77040.094	Yes	64	154208.187	154691.815	154488.429	1183.7999 (p ≤ 0.0001)	0.740
6	−76551.264	Yes	77	153256.527	153838.392	153593.693	969.853 (p < 0.0001)	0.703

Note: AIC: Akaike Information Criterion; BIC: Bayesian Information Criterion; SAIC: Sample-size Adjusted Information Criterion; LMR-LT: Lo-Mendell-Rubin Adjusted Likelihood Ratio Test.

### 3.4. Latent classes

**Figure 1.**
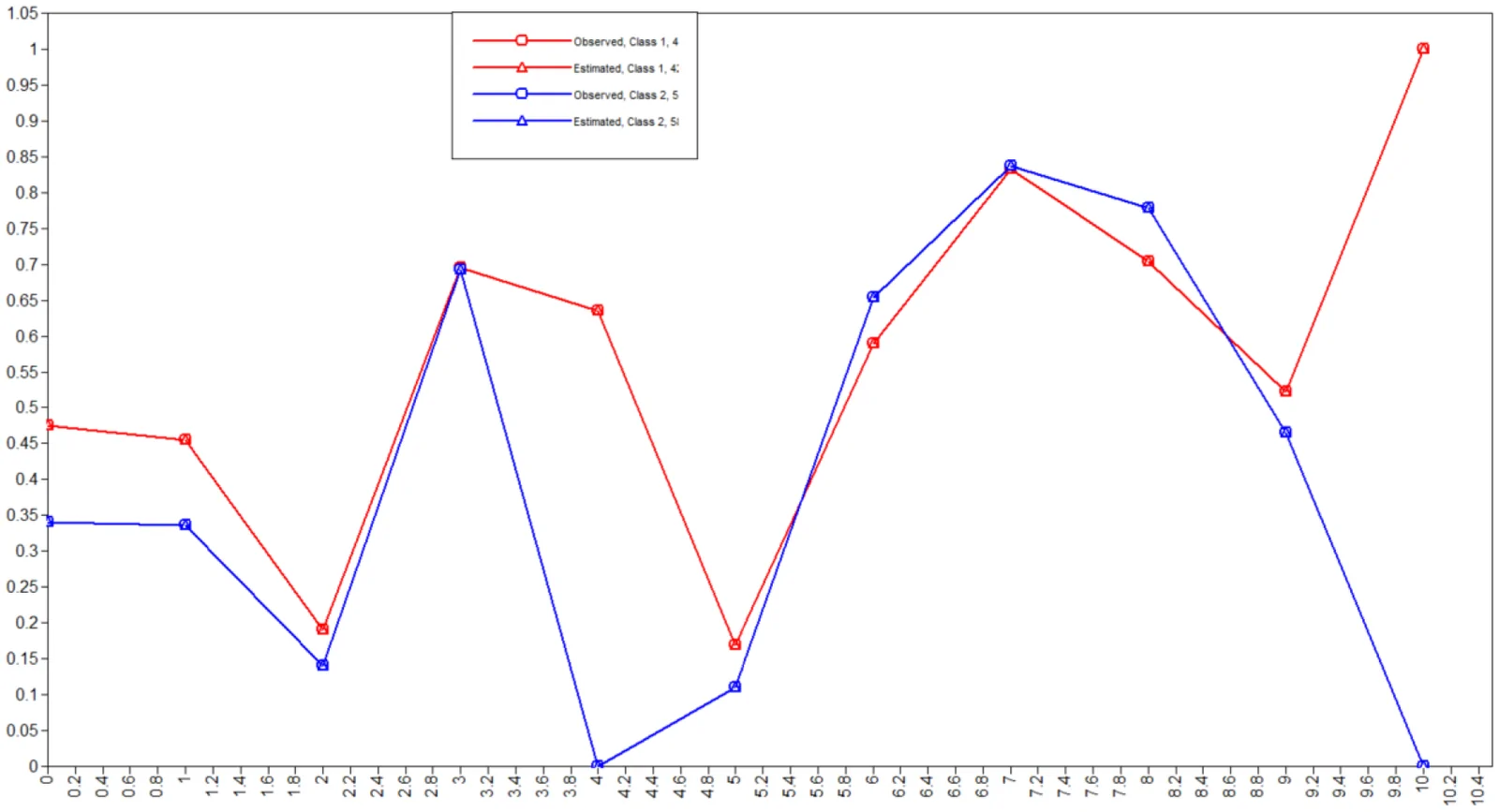
Plot of a two class LCA.

Table 4 presents the item-response probabilities for each latent class. Latent Class 1 represents approximately 58.5% of the North Carolina adult population. Individuals in this class had a lower probability of endorsing multiple health-related risk factors compared to Class 2. Specifically, the likelihood of having hypertension (34.1%) and high cholesterol (33.6%) was lower in this group. Nearly all individuals in Class 1 were non-smokers (99.98%), and the prevalence of current or former smoking was negligible. Members of this class reported a slightly higher prevalence of physical activity (77.8%) and fruit consumption (65.2%). However, the majority were still classified as overweight or obese (69.2%) and reported moderate alcohol use. Based on these characteristics, this class was named “Moderate drinking, overweight, non-smokers”. In contrast, Latent Class 2 is composed of 41.5% of the sample and had consistently higher probabilities of behavioral and chronic disease prevalence compared to Class 1. In this group, the prevalence of hypertension (47.5%), high cholesterol (45.4%), and diabetes (17.0%) was higher. Smoking was a defining feature of this class, with 36.5% identifying as current smokers and 63.5% as former smokers; no individuals in this class were non-smokers. Additionally, members of Class 2 were more likely to report binge drinking (16.8%), lower levels of physical activity (70.4%), and slightly lower fruit consumption (58.9%) compared to Class 1. Given this clustering of behavioral and chronic health risks, this class was named “High behavioral and chronic risk profile” (Figure 1).

**Table 4.** Prevalence and item-response probabilities of health-related risk factors by latent class membership.

	**Latent class 1** **“Moderate drinking overweight non-smokers”**	**Latent class 2** **“High behavioral and chronic risk profile”**
**Prevalence of latent class**	58.55	41.45
**Sample size**	8279	5860
**Item-response probabilities of endorsing a “yes” response**		
**% of adults who have been told they have high blood pressure**	34.1	47.5
**% of adults who have elevated cholesterol**	33.6	45.4
**% of adults who endorse the history of diabetes**	12.2	17.0
**% of overweight or obese**	**69.2**	**69.5**
**Smoking status**		
% Current smokers	0.01	36.5
% Former smokers	0.01	**63.5**
% Never smokers	**99.98**	0.00
**% Binge drinkers**	11.10	16.8
**% who consume Fruit 1 or more times per day**	**65.2**	**58.9**
**% who consume Vegetables 1 or more times per day**	**83.7**	**83.2**
**% who endorse Physical activity/exercise during the past 30 days**	**77.8**	**70.4**
**% of adults who reported at least one drink in the past 30 days**	46.4	**52.2**
**% of adults who have smoked at least 100 cigarettes in your entire life**	0.01	**99.99**

Note: The greater probabilities appear in bold font to highlight the overall pattern.

### 3.5. Multivariable regression analysis

To examine the demographic predictors of latent class membership, multi-logistic and multinomial logistic regression analyses were performed, and used the “Moderate drinking, overweight, non-smokers” (Class 1) as a reference.

### 3.6. Sociodemographic/Socioeconomic predictors of high-risk class membership

Table 5 shows the results of the multi-logistic regression analyses which examined the sociodemographic and socioeconomic predictors of high-risk latent class membership. Compared to males, females had significantly lower odds of being in the high-risk class (OR = 0.60, 95% CI: 0.54–0.67). Age was positively associated with high-risk class membership, with the participants aged 40–59 years having 68% higher odds (OR = 1.68, 95% CI: 1.48–1.90) and those aged 60 years and above having 70% higher odds (OR = 1.70, 95% CI: 1.49–1.93) of being in the high-risk class compared to the participants aged 18–39 years. Race and ethnicity showed a significant association with latent class membership. The non-Hispanic Black participants were 41% less likely to belong to the high-risk class compared to the non-Hispanic White participants (OR = 0.59, 95% CI: 0.52–0.68). Similarly, the non-Hispanic participants of other races had 39% lower odds (OR = 0.61, 95% CI: 0.47–0.78), and the Hispanic participants had 73% lower odds (OR = 0.27, 95% CI: 0.21–0.34) of being in the high-risk class compared to the non-Hispanic White participants. The multiracial, non-Hispanic participants did not show a statistically significant difference in high-risk class membership (OR = 0.95, 95% CI: 0.64–1.43). The participants with higher income levels were less likely to be in the high-risk class. The participants who earned $50,000 to <$100,000 had 30% lower odds (OR = 0.70, 95% CI: 0.57–0.85), those who earned $100,000 to <$200,000 had 55% lower odds (OR = 0.45, 95% CI: 0.34–0.59), and those who earned $200,000 or more had 46% lower odds (OR = 0.54, 95% CI: 0.35–0.83) of being in the high-risk class compared to those who earned less than $15,000. Adults who attended college or technical school were 22% less likely to be in the high-risk class (OR = 0.78, 95% CI: 0.63–0.98), while those who graduated from college or technical school were 65% less likely to be in the high-risk class (OR = 0.35, 95% CI: 0.28–0.44) compared to the participants who did not graduate high school. Graduating high school alone showed a marginal, non-significant reduction in high-risk class membership odds (OR = 0.83, 95% CI: 0.67–1.03).

### 3.7. Association of latent class membership and health outcomes [cardiovascular disease status (MI/CHD), self-reported health status, physically unhealthy days and mentally unhealthy days]

Table 6 presents the adjusted associations between latent class membership and two health outcomes: CVD and self-reported health status. Adults in the high-risk class were significantly more likely to report CVD compared to those in the moderate drinking overweight non-smokers class. The adjusted analyses showed that the participants in the high-risk class had nearly two times higher odds of reporting CVD (OR = 1.93, 95% CI = 1.60–2.34). Being stratified by age, the association was strongest among the participants aged 18–39 years, where the odds of reporting CVD were nearly seven times higher in the high-risk class compared to the reference group (OR = 6.84, 95% CI = 2.79–16.72). Among those aged 60 years and above, the odds were also significantly elevated (OR = 1.54, 95% CI = 1.23–1.93), while the association among the participants aged 40–59 years was not statistically significant (OR = 1.37, 95% CI = 0.94–2.02). For self-reported general health, the participants in the high-risk class were significantly more likely to report fair or poor health than the reference group. The adjusted analyses showed that the participants in the high-risk class had 69% higher odds of reporting worse health (OR = 1.69, 95% CI = 1.46–1.96). Being stratified by age, the association remained significant across all age groups, with the strongest association observed among the participants aged 18–39 years (OR = 1.79, 95% CI = 1.28–2.50). Among the participants aged 40–59 years, the odds of reporting worse health were 1.58 times higher in the high-risk class (OR = 1.58, 95% CI = 1.23–2.03), while the odds were slightly lower but still statistically significant among those aged 60 years and above (OR = 1.39, 95% CI = 1.13–1.71) (Table 6).

**Table 5.** Association of sociodemographic characteristics with the likelihood of belonging to the high behavioral and chronic risk profile latent class (vs. moderate drinking, overweight, non-smokers).

**Sociodemographic variables**	**Beta estimate**	**OR (95% CI)**	**P-value**
**Sex**			0.0002
Male (Ref)		1.00	
Female	−0.51	0.60 (0.54–0.67)	<0.0001
**Age group**			<0.0001
18–39 years (Ref)		1.00	
40–59 years	0.52	1.68 (1.48–1.90)	<0.0001
60 years and above	0.53	1.70 (1.49–1.93)	<0.0001
**Race/Ethnicity**			<0.0001
White, non-Hispanic (Ref)		1.00	
Black, non-Hispanic	−0.57	0.59 (0.52–0.68)	<0.0001
Other Race, non-Hispanic	−0.5	0.61 (0.47–0.78)	<0.0001
Multiracial, non-Hispanic	−0.05	0.95 (0.64–1.43)	0.8191
Hispanic	−1.32	0.27 (0.21–0.34)	<0.0001
**Household income**			<0.0001
<$15,000 (Ref)		1.00	
$15,000 to <$25,000	−0.02	0.98 (0.80–1.21)	0.8703
$25,000 to <$35,000	−0.08	0.92 (0.74–1.15)	0.4719
$35,000 to <$50,000	−0.26	0.77 (0.62–0.96)	0.0193
$50,000 to <$100,000	−0.36	0.70 (0.57–0.85)	0.0004
$100,000 to <$200,000	−0.8	0.45 (0.34–0.59)	<0.0001
≥$200,000	−0.62	0.54 (0.35–0.83)	0.0048
**Educational attainment**			<0.0001
Did not graduate high school (Ref)	1.00	
Graduated high school	−0.19	0.83 (0.67–1.03)	0.0900
Attended college/technical school	−0.25	0.78 (0.63–0.98)	0.0288
Graduated from college/technical school	−1.06	0.35 (0.28–0.44)	<0.0001

**Table 6.** Adjusted logistic regression of the association between latent class membership and cardiovascular disease and self-reported health status.

	**CVD (Adjusted OR, 95% CI)**	**Self-Reported Health (Adjusted OR, 95% CI)**
**Total population**		
	Adjusted^a^	Adjusted^a^
Moderate drinking overweight non-smokers	Ref	Ref
High behavioral and chronic risk profile	1.93 (1.60–2.34)	1.69 (1.46–1.96)
**18–39 years**		
	Adjusted^a^	Adjusted^a^
Moderate drinking overweight non-smokers	Ref	Ref
High behavioral and chronic risk profile	6.84(2.79–16.72)	1.79 (1.28–2.50)
**40–59 years**		
	Adjusted^a^	Adjusted^a^
Moderate drinking overweight non-smokers	Ref	Ref
High behavioral and chronic risk profile	1.37 (0.94–2.02)	1.58 (1.23–2.03)
**60 years and above**		
	Adjusted^a^	Adjusted^a^
Moderate drinking overweight non-smokers	Ref	Ref
High behavioral and chronic risk profile	1.54 (1.23–1.93)	1.39 (1.13–1.71)

Note: ^a^Model for the total population were adjusted for age, sex, race/ethnicity, educational attainment, and household income. Models stratified by age group were adjusted for sex, race/ethnicity, educational attainment, and household income.

In terms of physical health status, the participants in the high-risk class had higher odds of reporting both moderate and severe physical health difficulties compared to the reference group (Table 7). Overall, the odds of reporting 14+ days of poor physical health (compared to reporting zero days) were significantly elevated in the high-risk class (OR = 1.82, 95% CI = 1.55–2.15) compared to the low-risk class, with similar patterns observed across age groups. For example, the younger participants (18–39 years) in the high-risk class (compared to the lower risk class) were over twice as likely to report 14+ days of poor physical health (OR = 2.32, 95% CI = 1.58–3.40). The odds of reporting 14+ days of poor physical health (compared to 0 days of poor physical health) in the high-risk group (compared to the lower risk group) were significantly elevated among the participants aged 40–59 years and those aged 60 years and above (OR = 1.62, 95% CI = 1.23–2.14, OR = 1.41, 95% CI = 1.12–1.79, respectively). For mental health outcome, the participants in the high-risk class were significantly more likely to report poor mental health days (14+ days of poor mental health vs 0 days of poor mental health) compared to those in the lower risk class. Overall, the odds of reporting 14+ days of poor mental health (compared to 0 poor mental health days) were nearly 1.7 times higher in the high-risk class (OR = 1.68, 95% CI = 1.43–1.97) compared to the lower risk class. Age-stratified analyses showed the strongest association among the younger participants aged 18–39 years, where the odds were over twice as high in the high-risk class (OR = 2.12, 95% CI = 1.60–2.81) compared to the lower risk class. The association remained significant for the participants aged 40–59 years (OR = 1.77, 95% CI = 1.37–2.29) and those aged 60 years and above (OR = 1.47, 95% CI = 1.09–1.97).

**Table 7.** Adjusted multinomial logistic regression of the association of latent class membership and physical and mental health days.

	**Outcome: Physical health status**		**Outcome: Mental health status**	
	**1–13 days vs. 0 days^a^**	**14+ days vs. 0 days^a^**	**1–13 days vs. 0 days^a^**	**14+ days vs. 0 days^a^**
**Total population^a^**				
Moderate drinking overweight non-smokers	Ref	Ref	Ref	Ref
High behavioral and chronic risk profile	1.11 (0.98–1.26)	1.82 (1.55–2.15)	0.980 (0.86–1.11)	1.68 (1.43–1.97)
**18–39 years^a^**				
Moderate drinking overweight non-smokers	Ref	Ref	Ref	Ref
High behavioral and chronic risk profile	1.02 (0.82–1.29)	2.32 (1.58–3.40)	1.12 (0.90–1.39)	2.12 (1.60–2.81)
**40–59 years^a^**				
Moderate drinking overweight non-smokers	Ref	Ref	Ref	Ref
High behavioral and chronic risk profile	1.22 (0.98–1.52)	1.62 (1.23–2.14)	1.26 (1.02–1.56)	1.77(1.37–2.29)
**60 years and above^a^**				
Moderate drinking overweight non-smokers	Ref	Ref	Ref	Ref
High behavioral and chronic risk profile	1.16 (0.94–1.43)	1.41 (1.12–1.79)	1.03 (0.80–1.32)	1.47 (1.09–1.97)

Note: ^a^Models for the total population were adjusted for age, sex, race/ethnicity, educational attainment, and household income. Models stratified by age group were adjusted for sex, race/ethnicity, educational attainment, and household income.

## 4. Discussion

Our study explores the association between latent class membership and health outcomes by deriving clusters of health-related risk factors. These findings are important to understand and address health disparities in North Carolina. Consistent with the syndemic framework, our analysis recognizes that risk behaviors and chronic health conditions often cluster together within specific populations, interact synergistically, and contribute to cumulative health outcome burdens.

We derived two distinct latent classes of health-related risk factors among NC adults—moderate drinking overweight non-smokers and high behavioral and chronic risk profiles—characterized by differences in smoking, drinking, and chronic disease profiles. Notably, the high behavioral and chronic risk profile class had a higher prevalence of current and former smoking, binge drinking, and chronic conditions such as hypertension, high cholesterol, and diabetes compared to the moderate drinking overweight non-smokers class. Prior studies primarily focused on solely clustering behavioral risk factors [10]–[14],[30]–[34]; however, our inclusion of both behavioral and chronic health risks was grounded in recognizing that these conditions do not merely result from health behaviors but often co-occur and reinforce one another over time. This approach aligns with the syndemic theory, which posits that interconnected and mutually reinforcing health conditions tend to cluster within socioeconomically and structurally disadvantaged populations [27],[28].

Our findings align with prior research on clustering patterns of risk behaviors. For example, in their systematic review on clustering and co-occurrence of multiple risk behaviors, Meader et al. (2016) reported alcohol use and smoking clustering among adults independent of the utilized alcohol use measure [10]. They reported consistent findings from several countries, including the United States, Netherlands, and Hong Kong, where alcohol and smoking had the strongest evidence of clustering of all the risk behaviors examined [10]. Our results similarly demonstrate that smoking and alcohol consumption were primary distinguishing factors between latent classes. Although physical activity and fruit and vegetable intake were more favorable in the moderate drinking overweight non-smokers class, these behaviors did not differ as markedly as smoking and drinking between classes. Dodd et al. (2010) identified three clusters among UK adults: an unhealthy/high-risk profile (consisting of low fruit and vegetable intake, lack of physical activity, and high stress), a moderately healthy/moderate risk profile (consisting of greater prevalence of smoking, low fruit, and vegetable intake alongside more physical activity), and a healthy/low-risk profile (low prevalence of smoking, higher fruit and vegetable intake, and physical activity) [11]. Our study findings are distinct from their findings and could reflect unique population-level or state-level characteristics specific to North Carolina. Factors such as local health policies, cultural norms around smoking and alcohol use, and demographic differences might contribute to the distinct clustering of risk behaviors observed in our study.

Our study examined associations between latent class membership and key health outcomes. Adults in the higher risk profile class were significantly more likely to report adverse health outcomes compared to the moderate drinking overweight non-smokers class. Specifically, the odds of reporting CVD were nearly two times higher in the high-risk class. This finding is similar to one study that examined the clustering of cardiovascular disease risk factors and found clusters, such as smoking and hypertension, that contributed to the highest risk of CVD and all-cause mortality [47]. Using an LCA, a UK Biobank study found that multiple lifestyle risk behavior clustering (physically inactive, poor fruit & vegetable intake, high alcohol intake, and prolonged sitting) significantly increased both CVD risk and the odds of a CVD diagnosis. Specifically, individuals with three co-occurring behaviors had over 25 times the odds of having CVD compared to those with only two risk factors [48]. One study using data from the 2003 BRFSS found that adults with four or more cardiovascular risk factors (smoking, obesity, hypertension, high cholesterol, and diabetes) had 14 times higher odds of reporting poor or fair health and significantly more physically and mentally unhealthy days compared to those with no risk factors [49]. This reinforces our finding that the elevated odds of poor self-reported health and poor physical and poor mental health status observed in our high-risk class likely reflect the cumulative burden of co-occurring behaviors and chronic conditions.

Stratified analyses by age categories revealed that these associations significantly varied across age groups. Among the young adults aged 18–39 years, membership in the higher-risk class was associated with over six times the odds of reporting CVD compared to the lower-risk class. This striking association shows the vulnerability of younger adults to the effects of engaging in multiple health risk behaviors and living with early-onset chronic conditions. Indeed, an early initiation of smoking and excessive alcohol use, compounded by hypertension and diabetes, may accelerate disease progression and increase the lifetime cardiovascular risk. For example, a prospective study of young adults with stage 1 hypertension found that combined smoking and alcohol use more than quadrupled the risk of major cardiovascular events, with the highest risk among heavy users of both [50]. Similarly, diabetes and hypertension commonly co-occur and jointly contribute to an earlier onset and greater severity of cardiovascular complications through their shared mechanisms of inflammation and vascular damage [51]. In contrast, the association between latent class membership and CVD was less pronounced in the older adults, possibly due to the cumulative impact of age-related health risks, such as hypertension, diabetes, and multimorbidity, which may overshadow the effects of smoking and drinking behaviors. This pattern is consistent with findings from a longitudinal study by Suhag et al. (2024), which showed that among older adults, clusters characterized by physical inactivity and socioeconomic disadvantage—not necessarily smoking or drinking—were associated with the highest rates of multimorbidity and complex multimorbidity [31]. Their result suggests that by an older age, the burden of other chronic conditions may dominate the health risk landscape, thus diminishing the relative contribution of behavioral risks. Furthermore, the weaker associations observed in older adults may reflect a survivor bias, as individuals with the most severe risk profiles may not survive to a later life [52]. Longitudinal studies are needed to further explore these age-related dynamics and clarify how the cumulative burden of behaviors and conditions shape cardiovascular risk over the life course.

Beyond cardiovascular outcomes, individuals in the high-risk class demonstrated a significantly poorer self-reported physical health compared to those in the moderate drinking, overweight, non-smokers class. While no prior study has evaluated the precise combination of risk factors analyzed in our study, our findings are consistent with the broader literature on the impact of behavioral risk factors on self-reported general and physical health. Using data from NHANES 2009–2016, Ware et al. (2022) applied an LCA and identified two distinct groups based on adherence to healthy behaviors, specifically smoking status, physical activity, and diet quality [13]. They found that individuals in the class characterized by fewer healthy behaviors were significantly less likely to report good, very good, or excellent self-rated health compared to those in the healthier behavior class. Oftedal et al. (2019) identified distinct patterns of co-occurring health behaviors among Australian adults and reported that individuals in high-risk behavior classes (defined by smoking, poor diet, physical inactivity, and high alcohol intake) were more likely to report poorer self-rated health [30].

We found that belonging to the high-risk group was also associated with higher odds of reporting a poorer mental health status. El Ansari et al. (2024) identified three behavioral risk clusters (healthy group, smokers, nonsmokers but problematic drinkers) among Finnish university students, and reported that students in the “smokers” and “non-smokers but problematic drinkers” cluster were significantly more likely to experience higher depressive symptoms compared to the “healthy group”, while students in the “smoker’ cluster were more likely to report higher stress [33]. Vermeulen-Smit et al. (2015) identified four behavioral classes, which included a “most healthy” class and three unhealthy classes characterized by smoking, heavy drinking, physical inactivity, and poor diet. Adults in all three unhealthy classes had approximately double the risk of depression and higher rates of other mental disorders compared to the healthy class [34]. Champion et al. (2020) identified three latent classes of risk behaviors among young Australian adults: “moderate risk” (52%), “inactive, non-smokers” (24%), and “smokers and binge drinkers” (24%) [14]. The mental health symptoms were significantly higher in the “smokers and binge drinkers” class, who reported greater psychological distress, depression, and anxiety compared to the moderate risk group [14]. Additionally, the “inactive, non-smokers” class showed elevated depression scores. The co-occurrence of these risk behaviors may exert a cumulative and synergistic effect on mental health through shared neurobiological pathways. Evidence suggests that nicotine dependence disrupts the serotonergic and dopaminergic systems and increases the vulnerability to anxiety and depression [53]. Excessive alcohol consumption may further impair mood regulation by inducing oxidative stress and neuroinflammation [54]. The clustering of these behaviors likely compounds their adverse impact on mental health and illustrates the need for integrated intervention strategies which target multiple behavioral risk factors.

Our study findings offer some implications for public health intervention and policy in North Carolina and highlight the need for concurrent syndemic-informed strategies to address behavioral and chronic health risks. North Carolina can leverage existing programs and policies to target these risk clusters. QuitlineNC provides free quit-smoking services and could be expanded to better reach people at a higher risk [55]. Its personalized tools, such as quit coaching and nicotine replacement therapies, may help adults who are trying to quit or are at risk of relapse. In addition, North Carolina's Smoke-Free NC initiative, which promotes smoke-free spaces, can help limit secondhand smoke exposure and reduce the related health risks [56]. Community engagement programs such as “Talk It Up. Lock It Up!” and “Sticker Shock,” which are part of North Carolina's Preventing Underage Drinking Initiative (NCPUDi), can be strategically expanded to deter alcohol misuse among young adults [57]. State initiatives such as the NC Tobacco Prevention and Control Branch's health equity efforts [58] and the Alcohol/Drug Council of North Carolina's services [59] should continue to be leveraged to deliver culturally tailored interventions to those most at risk. Preventive health screening should be a routine component of primary care visits, irrespective of the patient's insurance type. Information integration on effective screening and referral systems between public health, community, and tertiary hospital systems could offer a cost-effective and scalable early intervention and follow-up strategy to prevent relapse. Additionally, Recovery Support Services in North Carolina could be critical in providing follow-up care support for individuals transitioning from high-risk behaviors to healthier lifestyles [60]. However, the sustainability of these programs depends on consistent state and county-level funding and willpower, as well as efforts to expand their capacity and strengthen their infrastructure. The need for ongoing evaluation, monitoring, and retooling of these programs is equally important to ensure that these initiatives and programs continue to meet the community needs, achieve their intended goals, and adapt to evolving public health challenges.

One strength of our study is using an LCA to identify meaningful subgroups based on co-occurring behavioral and chronic health risks. Using a large representative sample with an adjustment for potential confounders also enhances the generalizability of our findings to the state. Some limitations should be kept in mind. The cross-sectional design limits the ability to draw causal inferences or to assess the dynamic interactions among risk factors over time. Prospective longitudinal studies are needed to fully capture the temporal and reciprocal relationships between clustered risk factors and health outcomes. The self-reported format of the survey may introduce recall and reporting biases, especially for sensitive behaviors such as smoking and alcohol consumption. Another potential limitation is the misclassification of individuals within latent classes due to measurement errors or an unobserved heterogeneity. Latent class may oversimplify the complexity of individual risk profiles. More so, due to the cross-sectional design, the temporal progression from gestational diabetes or prediabetes to type 2 diabetes cannot be determined. These cases (n < 150 per wave) were excluded to maintain clarity in the binary classification of diabetes consistent with latent class modeling. Furthermore, given that North Carolina has a higher prevalence of cardiovascular and behavioral risk factors compared to some US states, the generalizability of our latent class structures and their associations with outcomes to other regions may be limited. Finally, while our analytic approach aligns with the syndemic theory to identify clusters of co-occurring behavioral and chronic health risks and their associations with adverse health outcomes, the lack of a direct assessment of structural determinants limits our ability to fully operationalize all aspects of this framework in our modelling.

## 5. Conclusions

We observed significant associations between latent class membership and health outcomes, with individuals in the higher-risk group experiencing substantially worse cardiovascular, physical, and mental health outcomes compared to those in the lower-risk group. These findings reinforce the need for targeted public health interventions and policies that address the co-occurrence of behavioral and chronic disease risk factors, particularly among young adults. Tailored strategies, such as integrating smoking cessation programs and alcohol reduction initiatives into community-based public health efforts, may help mitigate the adverse health consequences of these behaviors. Routine screening for hypertension, diabetes, and hypercholesterolemia during physician visits, which is in line with the United States Preventive Services Task Force (USPSTF) guidelines, should be complemented by an increased attention to behavioral risks, especially in younger adults. Aligning behavioral health interventions with preventive care strategies has the potential to reduce individual health risks and improve population health outcomes in order to achieve the Healthy People 2030 target. Future research should explore the longitudinal impact of these high-risk profiles on morbidity and mortality within the state.

## Use of AI tools declaration

The authors declare they have not used Artificial Intelligence (AI) tools in the creation of this article.

## References

[1] Bauer UE, Briss PA, Goodman RA (2014). Prevention of chronic disease in the 21st century: elimination of the leading preventable causes of premature death and disability in the USA. Lancet.

[2] Ng R, Sutradhar R, Yao Z (2020). Smoking, drinking, diet and physical activity-modifiable lifestyle risk factors and their associations with age to first chronic disease. Int J Epidemiol.

[3] Mukamal KJ (2006). The effects of smoking and drinking on cardiovascular disease and risk factors. Alcohol Res Health.

[4] Oster H, Chaves I (2023). Effects of healthy lifestyles on chronic diseases: diet, sleep and exercise. Nutrients.

[5] Linardakis M, Papadaki A, Smpokos E (2015). Association of behavioral risk factors for chronic diseases with physical and mental health in European adults aged 50 years or older, 2004–2005. Prev Chronic Dis.

[6] Centers for Disease Control and Prevention (2024). National Center for Health Statistics, Mortality Data on CDC WONDER, All Ages Deaths by Multiple Cause of Death 2018–2022.

[7] Martin SS, Aday AW, Almarzooq ZI (2024). 2024 heart disease and stroke statistics: a report of US and global data from the American Heart Association. Circulation.

[8] Justus-Warren Heart Disease and Stroke Prevention Task Force (2020). The Burden of Cardiovascular Disease in North Carolina.

[9] Eat Smart, Move More NC (2019). Heart and Blood Vessel Disease Fact Sheet. Community and Clinical Connections for Prevention and Health Branch, North Carolina Division of Public Health.

[10] Meader N, King K, Moe-Byrne T (2016). A systematic review on the clustering and co-occurrence of multiple risk behaviours. BMC Public Health.

[11] Dodd LJ, Al-Nakeeb Y, Nevill A (2010). Lifestyle risk factors of students: a cluster analytical approach. Prev Med.

[12] Spring B, Moller AC, Coons MJ (2012). Multiple health behaviours: overview and implications. J Public Health (Oxf).

[13] Ware D, Landy DC, Rabil A (2022). Interrelationships between self-reported physical health and health behaviors among healthy US adults: From the NHANES 2009–2016. Public Health Pract (Oxf).

[14] Champion KE, Mather M, Spring B (2018). Clustering of multiple risk behaviors among a sample of 18-year-old Australians and associations with mental health outcomes: a latent class analysis. Front Public Health.

[15] County Health Rankings & Roadmaps (2021). 2021 County Health Rankings for North Carolina: Measures and national/state results.

[16] Centers for Disease Control and Prevention (2018). North Carolina State Action Guide on Fruits and Vegetables.

[17] Community and Clinical Connections for Prevention and Health (2024). High blood pressure in North Carolina fact sheet.

[18] Diabetes Management North Carolina (2021). Type 2 Diabetes in North Carolina fact sheet.

[19] America's Health Rankings, United Health Foundation (2023). High cholesterol – North Carolina.

[20] Bhatti Z, Salek M, Finlay A (2011). Chronic diseases influence major life changing decisions: a new domain in quality of life research. J R Soc Med.

[21] American Action Forum (2023). Understanding the connections between chronic disease and individual-level risk factors.

[22] Davis VH, Zhang G, Patel MR (2025). Chronic disease and future perceptions of financial control: results from the Midlife in the United States cohort study. Med Care.

[23] Hall PA, Marteau TM (2014). Executive function in the context of chronic disease prevention: theory, research and practice. Prev Med.

[24] Reimann Z, Miller JR, Dahle KM (2018). Executive functions and health behaviors associated with the leading causes of death in the United States: a systematic review. J Health Psychol.

[25] Pirrone I, Dieleman M, Reis R (2021). Syndemic contexts: findings from a review of research on non-communicable diseases and interviews with experts. Glob Health Action.

[26] Hossain MM, Saha N, Rodela TT (2022). Global research on syndemics: a meta-knowledge analysis (2001–2020). F1000Res.

[27] Sharma A (2017). Syndemics: health in context. Lancet.

[28] Singer M, Bulled N, Ostrach B (2017). Syndemics and the biosocial conception of health. Lancet.

[29] Collins LM, Lanza ST (2009). Latent Class and Latent Transition Analysis: With Applications in the Social, Behavioral, and Health Sciences.

[30] Oftedal S, Kolt GS, Holliday EG (2019). Associations of health-behavior patterns, mental health and self-rated health. Prev Med.

[31] Suhag A, Webb TL, Holmes J (2024). Longitudinal clustering of health behaviours and their association with multimorbidity in older adults in England: a latent class analysis. PLoS One.

[32] Hutchesson MJ, Duncan MJ, Oftedal S (2021). Latent class analysis of multiple health risk behaviors among Australian university students and associations with psychological distress. Nutrients.

[33] El Ansari W, Sebena R, El-Ansari K (2024). Clusters of lifestyle behavioral risk factors and their associations with depressive symptoms and stress: evidence from students at a university in Finland. BMC Public Health.

[34] Vermeulen-Smit E, Ten Have M, Van Laar M (2015). Clustering of health risk behaviours and the relationship with mental disorders. J Affect Disord.

[35] Krauth SJ, Steell L, Ahmed S (2024). Association of latent class analysis-derived multimorbidity clusters with adverse health outcomes in patients with multiple long-term conditions: comparative results across three UK cohorts. EClinicalMedicine.

[36] Olaya B, Moneta MV, Caballero F (2017). Latent class analysis of multimorbidity patterns and associated outcomes in Spanish older adults: a prospective cohort study. BMC Geriatr.

[37] Shri N, Singh S, Singh SK (2024). Latent class analysis of chronic disease co-occurrence, clustering and their determinants in India using Study on Global AGEing and Adult Health (SAGE) India Wave-2. J Glob Health.

[38] Moriarty DG, Zack MM, Kobau R (2003). The Centers for Disease Control and Prevention's Healthy Days Measures—population tracking of perceived physical and mental health over time. Health Qual Life Outcomes.

[39] NCDHHS, Division of Public Health, NC State Center for Health Statistics (2021). SCHS: BRFSS Annual Survey Results.

[40] NCDHHS, Division of Public Health, NC State Center for Health Statistics (2017). Behavioral Risk Factor Surveillance System (BRFSS) technical notes 2017.

[41] NCDHHS, Division of Public Health, NC State Center for Health Statistics (2019). Behavioral Risk Factor Surveillance System (BRFSS) technical notes 2019.

[42] NCDHHS, Division of Public Health, NC State Center for Health Statistics (2021). Behavioral Risk Factor Surveillance System (BRFSS) technical notes 2021.

[43] American Association for Public Opinion Research (2023). Standard Definitions: Final Dispositions of Case Codes and Outcome Rates for Surveys.

[44] Tabák AG, Herder C, Rathmann W (2012). Prediabetes: a high-risk state for diabetes development. Lancet.

[45] Ogbu CE, Ravilla J, Okoli ML (2023). Association of depression, poor mental health status and asthma control patterns in US adults using a data-reductive latent class method. Cureus.

[46] Kankaraš M, Moors G, Vermunt JK (2018). Testing for measurement invariance with latent class analysis. Cross-Cultural Analysis.

[47] Holthuis EI, Visseren FLJ, Bots ML (2021). Risk factor clusters and cardiovascular disease in high-risk patients: the UCC-SMART study. Glob Heart.

[48] Tegegne TK, Islam SMS, Maddison R (2022). Effects of lifestyle risk behaviour clustering on cardiovascular disease among UK adults: latent class analysis with distal outcomes. Sci Rep.

[49] Li C, Ford ES, Mokdad AH (2008). Clustering of cardiovascular disease risk factors and health-related quality of life among US adults. Value Health.

[50] Palatini P, Fania C, Mos L (2017). Alcohol intake more than doubles the risk of early cardiovascular events in young hypertensive smokers. Am J Med.

[51] Petrie JR, Guzik TJ, Touyz RM (2018). Diabetes, hypertension, and cardiovascular disease: clinical insights and vascular mechanisms. Can J Cardiol.

[52] Lijfering WM, Cannegieter SC (2018). Nutrition and venous thrombosis: an exercise in thinking about survivor bias. Res Pract Thromb Haemost.

[53] Laviolette SR (2021). Molecular and neuronal mechanisms underlying the effects of adolescent nicotine exposure on anxiety and mood disorders. Neuropharmacology.

[54] Sanchez-Marin L, Pavon FJ, Decara J (2017). Effects of intermittent alcohol exposure on emotion and cognition: a potential role for the endogenous cannabinoid system and neuroinflammation. Front Behav Neurosci.

[55] North Carolina Department of Health and Human Services, QuitlineNC. [cited 2025 March 25].

[56] North Carolina Department of Health and Human Services, Smoke-Free NC.

[57] North Carolina Department of Health and Human Services, North Carolina Preventing Underage Drinking Initiative (NCPUDi).

[58] North Carolina Department of Health and Human Services, Tobacco Prevention and Control Branch.

[59] Alcohol/Drug Council of North Carolina.

[60] North Carolina Recovery Support Services.

